# Comprehensive Genomic Profiling in Advanced Non‐Small Cell Lung Cancer: A Real‐World Cohort Study in Finland

**DOI:** 10.1002/cam4.71250

**Published:** 2025-09-28

**Authors:** Kirsi Hormalainen, Kaisa Marttila, Matti Nykter, Toomas Uibu, Jarkko Ahvonen, Vidal Fey, Mauri Keinänen, Maarit Bärlund, Arja Jukkola

**Affiliations:** ^1^ Department of Respiratory Medicine Tampere University Hospital Tampere Finland; ^2^ Tays Cancer Centre Tampere University Hospital Tampere Finland; ^3^ Faculty of Medicine and Health Technology Tampere University Tampere Finland; ^4^ Department of Oncology Tampere University Hospital Tampere Finland; ^5^ Fimlab Laboratories Tampere Finland

**Keywords:** biomarker, comprehensive genomic profiling, non‐small cell lung cancer, targeted therapy

## Abstract

**Background:**

Non‐small cell lung cancer (NSCLC) is a disease with a low survival rate and poor prognosis. Targeted therapies have improved treatment outcomes as driver mutations have been identified, especially in adenocarcinomas. Comprehensive genomic profiling (CGP) provides insights into the genetic mutation profile of cancer and helps identify actionable mutations. The mutational landscape of cancer varies based on the patient's ethnic background, and there is limited information on the genetic profile of NSCLC within the Finnish population.

**Material and Methods:**

We analysed the genetic mutational profile of 96 advanced NSCLCs that underwent CGP between November 2021 and March 2023 at Tampere University Hospital. Additionally, we compared the genomic alterations in our cohort with those in the international datasets.

**Results:**

Clinically actionable alterations associated with a targeted therapy were identified in 45% of patients, including 63% of never‐smokers and 41% of ever‐smokers. The most common actionable alteration was KRAS G12C (18%), followed by EGFR alterations (14%). However, only 33% of the patients with an actionable alteration received targeted therapy. The median tumour mutational burden (TMB) was 5, with 31% of patients exhibiting a TMB greater than 10.

**Conclusions:**

CGP affects the treatment strategies for NSCLC. Nearly half of our entire cohort had a genetic alteration eligible for approved targeted therapies. Besides these findings, CGP provides additional data to assess treatment decisions and outcomes, including co‐occurring genetic alterations and TMB. In real‐world clinical practice, the practical application of this information can be restricted by the varying unavailability of optimal treatments.

**Abbreviations**
CGP: comprehensive genomic profiling
ESCAT: ESMO Scale for Clinical Actionability of molecular Targets
F1CDx: FoundationOne CDx
F1LCDx: FoundationOne Liquid CDx
MSKCC: Memorial Sloan Kettering Cancer Center
TKI: tyrosine kinase inhibitors
TMB: tumour mutational burden

## 1 Introduction

Lung cancer is the leading cause of cancer‐related mortality globally and in Finland, accounting for 19% of global and 18% of Finnish cancer deaths in 2022 [1, 2]. The five‐year relative survival rate for Finnish lung cancer patients in 2020–2022 was 17%, significantly lower than for all cancers (69% for men and 71% for women). This poor prognosis is mainly due to late‐stage diagnosis, with over 50% of Finnish non‐small cell lung cancer (NSCLC) patients diagnosed at an advanced stage (stage IV), limiting treatment options [3].

The prognosis for lung cancer has improved in recent years, partly due to the identification of several driver mutations and targeted therapies, which have transformed both treatment and patient outcomes [4]. Genetic mutations have been identified across all lung cancer subtypes, particularly in adenocarcinomas. Common driver mutations in adenocarcinomas include mutations in the EGFR, KRAS, ROS, and RET genes, for which targeted therapies are available [5].

Cancers of the same histological type can exhibit highly variable genetic profiles. Next‐generation sequencing (NGS) enables the simultaneous, cost‐effective analysis of multiple mutations across various genes using minimal sample material [6]. Comprehensive genomic profiling (CGP) further enhances this by analysing hundreds of gene markers simultaneously, identifying clinically relevant mutations that may be targeted with drug therapies or influence treatment outcomes [7].

Cancer mutation prevalence is influenced by the patient's ethnic background [8]. Notable differences have been observed regarding EGFR mutations, which have the highest prevalence in Southeast Asian populations and a significantly lower prevalence in Western countries. The Finnish population is genetically distinct from the broader European population, and previous genetic profiling of non‐metastatic lung cancer has revealed differences in the frequency of specific mutations [9]. However, to date, no studies have been published that specifically examine genetic findings in advanced lung cancer in the Finnish population.

In this study, we determined the distribution of genetic findings identified by the FoundationOne CDx gene panel in advanced NSCLC patients. The aim was to investigate whether the mutation profile in the Finnish dataset differs from that in the international datasets, thus providing new and valuable insights into genetic findings in advanced NSCLC within the Finnish population.

## 2 Material and Methods

### 2.1 Patients

We conducted a retrospective, observational study of all patients with non‐squamous NSCLC, as well as those with squamous cell lung cancer who had smoked less than 10 pack‐years, who underwent comprehensive genomic profiling between November 2021 and March 2023 at Tampere University Hospital. In November 2021, CGP was adopted for routine clinical use at Tampere University Hospital for the evaluation of patients with advanced NSCLC. CGP was intended for patients with suspected advanced‐stage NSCLC who were deemed eligible for anticancer drug therapy. The hospital serves approximately 9% of the Finnish population, and during the study period, CGP was performed on 96 NSCLC patients. We retrospectively reviewed electronic patient records to identify all individuals assessed for NSCLC at Tampere University Hospital between November 2021 and March 2023. All patients who underwent CGP were included in the study cohort. NSCLC diagnoses were made by institutional pathologists according to the 2021 WHO Classification of Thoracic Tumours [10], and tumours were staged according to the current 8th edition of the TNM classification. PD‐L1 status was determined through IHC performed on formalin‐fixed paraffin‐embedded (FFPE) tissue sections, with the use of SP263 PD‐L1 antibody (Roche‐Ventana).

The study was conducted in accordance with national legislation on the Secondary Use of Health and Social Data (552/2019), which permits the pseudonymised processing and access to personal health data collected as part of standard clinical practice for research purposes. The Wellbeing Services County of Pirkanmaa, as the data controller, granted permission for data access (permit number 2802/2023). In line with the guidelines of the Finnish National Board on Research Integrity (TENK), ethical review by a research ethics committee was not required for this registry‐ and archive‐based study [11]. As stipulated by the legislation, the reported results must be anonymised, and individual‐level data cannot be reported. Basic pseudonymised patient demographics and clinical information were gathered from electronic patient records, together with the CGP results. A waiver for informed consent was obtained in accordance with the legislation.

### 2.2 Next‐Generation Sequencing

The FoundationOne CDx (F1CDx: Foundation Medicine Inc., Cambridge, MA, USA) [12] comprehensive genomic profiling assay was utilised for NGS analysis. F1CDx was performed on FFPE tumour material from 93 patients using specimens containing at least 50 ng of DNA for library construction. F1CDx employs targeted high‐throughput hybridisation‐based capture technology to detect substitutions, insertion and deletion alterations (indels), and copy number alterations in 324 genes, along with select gene rearrangements and genomic signatures, including microsatellite instability (MSI) and tumour mutational burden (TMB) [12, 13]. FoundationOne Liquid CDx (F1LCDx) covers 324 genes, tumour mutational burden measured from blood (bTMB), and high microsatellite instability (MSI‐H) [14]. F1LCDx was used on plasma derived from peripheral whole blood for three patients whose tissue samples were not adequate for F1CDx.

### 2.3 Statistical Analyses

The frequencies of genetic variants between different groups were compared using the chi‐square test or Fisher's exact test. Linear correlations were assessed using Spearman's test. A *p*‐value of less than 0.05 was considered statistically significant. Statistical analyses were performed using SPSS (IBM, version 29.0.1.0). Variant prevalence was calculated and visualised using R (R Core Team, version 4.4.2).

## 3 Results

### 3.1 Patient Characteristics

Comprehensive genomic profiling using F1CDx or F1LCDx was conducted on 96 NSCLC patients. The median age was 73 years (range 46–91), with 65% being male. Nineteen patients (20%) in the cohort were never‐smokers, the majority of whom were female (68%, 13 out of 19). Adenocarcinoma was the most common tumour histology, accounting for 92% of patients. In accordance with institutional guidelines, four patients with squamous cell lung cancer and fewer than 10 pack‐years of smoking were also tested. Most patients had stage IV NSCLC (80%). Three patients had an immigrant background, while the remainder were of Finnish European descent. The clinical characteristics of the cohort are summarised in Table 1.

**TABLE 1:** Clinical characteristics of the cohort.

	Entire cohort, *n* = 96 (%)
Sex	
Male	62 (65)
Female	34 (35)
Smoking status	
Ever‐smoker	76 (79)
Never‐smoker	19 (20)
Missing	1 (1)
Histology	
Adenocarcinoma	88 (92)
Squamous cell carcinoma	4 (4)
Pleomorphic carcinoma	3 (3)
NSCLC NOS	1 (1)
Stage	
I–II	4 (4)
III	15 (16)
IV	77 (80)
Sample method	
Biopsy	47 (49)
Cytology	36 (38)
Resection	10 (10)
Liquid biopsy	3 (3)
PD‐L1 status	
< 1	31 (32)
1–49	33 (34)
≥ 50	28 (29)
Missing	4 (4)
ECOG	
0	9 (9)
1	28 (29)
2	32 (33)
3	25 (26)
4	2 (2)

Most patients were newly diagnosed, while thirteen had recurrent disease and had previously undergone treatment. Of these, ten had received surgical treatment, and in eight cases, NGS samples were taken from the original surgical specimen. None had received tyrosine kinase inhibitors (TKIs) prior to NGS analysis; however, two had received immuno‐oncological treatment—one with atezolizumab following prior platinum‐based chemotherapy and the other with nivolumab following chemoradiotherapy. For the remaining 88 patients, samples were collected from the most accessible site. One patient with recurrent disease had undergone chemoradiotherapy before NGS sampling. Forty‐six samples were taken from the primary tumour, while 39 were retrieved from metastatic sites. In total, 57 samples were histological, and 36 were cytological. Additionally, three liquid biopsies were collected from plasma derived from peripheral whole blood.

### 3.2 Mutational Profile of Tumours

**FIGURE 1:**
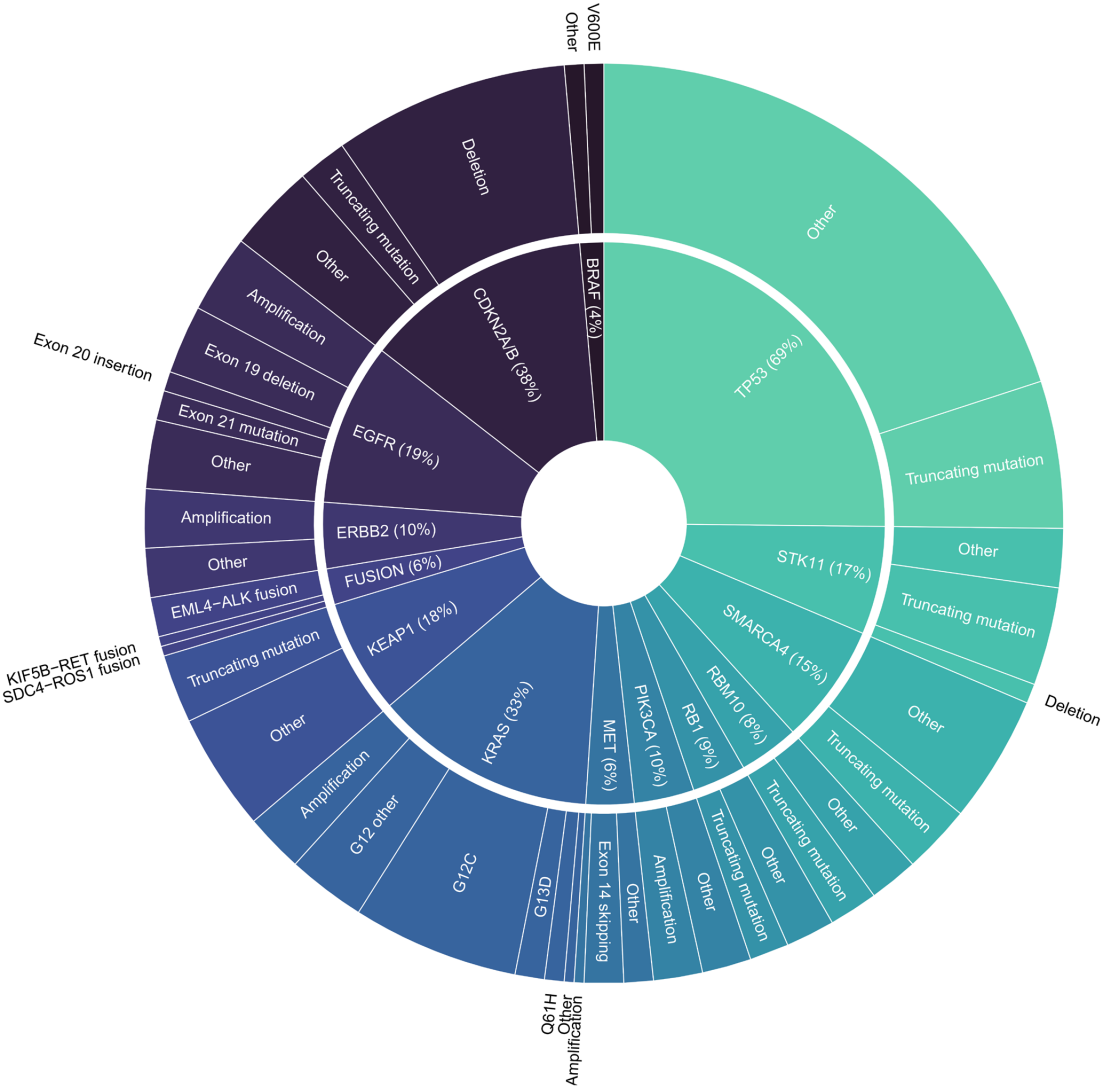
The genetic alterations most relevant in NSCLC.

Genetic alterations were identified in 256 genes. The most frequently mutated gene was *TP53*, with 69% of tumours exhibiting one or more alterations. This was followed by tumour suppressor gene *CDKN2A/B* (38%). The most commonly mutated oncogene was *KRAS*, altered in 33% of tumours. *RICTOR*, a core component of the oncogenic mTOR2 complex, was altered in 21% of tumours, as was the tumour suppressor gene *KMT2D*. Alterations in *EGFR* were found in 19% of tumours. This was followed by tumour suppressor genes *KEAP1* (18%), *STK11* (17%), and *MTAP* (17%). Less frequently, alterations were observed in the oncogenes *ERBB2* (10%), *MET* (6%), and *BRAF* (4%). The genes most relevant to NSCLC are illustrated in Figure 1.

Fusions were identified in seven patients (7.3%), including ALK in four patients and ROS1, RET, and ABL1 in one patient each. No FGFR or NTRK fusions were detected. EML4‐ALK fusions included three with Variant 1 and one with Variant 3a/b. One patient with an EML4‐ALK Variant 1 fusion also exhibited an ALK‐ZRANB3 non‐canonical fusion. The remaining fusions included one each of the SDC4‐ROS1, KIF5B‐RET, and ABL1‐TSC1 non‐canonical fusions. Clinically actionable gene alterations were classified using the ESMO Scale for Clinical Actionability of molecular Targets (ESCAT) classification system [15]. Actionable ESCAT level I/II alterations identified in our cohort are presented in Table 2.

**TABLE 2:** Targeted therapy for ESCAT level I/II alterations across the cohort (*n* = 96).

Gene	Alteration	No. of patients (%)	ESCAT score	Therapy	Approval or evidence
*ALK*	Fusion	4 (4.2)	I	1st‐ to 2nd‐generation ALK inhibitors Lorlatinib	EMA FDA
*ROS1*	Fusion	1 (1.0)	I	Crizotinib Entrectinib Repotrectinib^a^	EMA FDA
*RET*	Fusion	1 (1.0)	I	Pralsetinib Selpercatinib	EMA FDA
*EGFR*	Exon 19 deletion	7 (7.3)	I	1st‐to 3rd‐generation EGFR TKIs	EMA FDA
	Leu858Arg	2 (2.1)	I	Gefitinib Erlotinib Afatinib Osimertinib Dacomitinib Amivantamab + carboplatin + pemetrexed	EMA FDA
	Ser768Ile + Gly719Ser	1 (1.0)	I	Afatinib Osimertinib Erlotinib Gefitinib Dacomitinib	NCCN guidelines level 2A
	Leu861Gln + Leu833Phe	1 (1.0)	I	Afatinib Osimertinib Erlotinib Gefitinib Dacomitinib	NCCN guidelines level 2A
	Exon 20 insertion	2 (2.1)	I	Amivantamab Mobocertinib^a^	EMA FDA
*KRAS*	Gly12Cys	17 (17.7)	I	Sotorasib Adagrasib	EMA FDA
*MET*	Exon 14 skipping	4 (4.2)	I	Capmatinib Tepotinib Crizotinib^b^	EMA FDA NCCN guidelines level 2A
	Amplification	1 (1.0)	II	Capmatinib Crizotinib Tepotinib	NCCN guidelines level 2A
*BRAF*	Val600Glu	2 (2.1)	I	Dabrafenib + trametinib Encorafenib + binimetinib	EMA FDA
*ERBB2*	Exon 20 insertion	1 (1.0)	II	Fam‐trastuzumab deruxtecan Ado‐trastuzumab emtansine^b^	EMA FDA NCCN guidelines level 2A

Abbreviations: EMA, European Medicines Agency; ESCAT, ESMO Scale for Clinical Actionability of molecular Targets; FDA, U.S. Food and Drug Administration; TKI, tyrosine kinase inhibitor.

^a^ Only FDA approval.

^b^ NCCN guidelines treatment options, level 2A.

Alterations in the *EGFR* gene were identified in 18 patients (19%), with actionable ESCAT level I alterations detected in 13 (14%). TKI‐sensitising *EGFR* mutations were found in 11 patients, while *EGFR* exon 20 insertions were identified in two, as detailed in Table 2. The two patients with the Leu858Arg mutation had compound *EGFR* mutations, one with the Leu833Val mutation and the other with the Arg766His mutation. Two patients had rare *EGFR* mutations conveying sensitivity to second‐generation *EGFR* tyrosine kinase inhibitors: one with Ser768Ile and Gly719Ser, and the other with Leu861Gln and Leu833Phe. Eight *EGFR* amplifications were observed: four occurred alongside *EGFR* exon 19 deletions, one with an *EGFR* exon 20 insertion, and three independently. Additionally, two patients had *EGFR* variants of unknown significance: Tyr270Cys and Glu282Lys.

For *KRAS*, the most common genomic alteration was Gly12Cys, present in 53% of patients with any *KRAS* alteration, and classified as an ESCAT level I alteration. This was followed by Gly12Val (*n* = 5, 16%), Gly12Asp (*n* = 3, 9%), Gly13Asp (*n* = 3, 9%), Gln61His (*n* = 2, 6%), and Gly12Phe in one patient. Six *KRAS* amplifications were identified: three co‐occurring with Gly12Cys, one with Gly12Val, and two occurring independently. MET exon 14 skipping mutations were identified in four patients, one of whom also had a concurrent *MET* amplification. Additionally, a Leu1195Phe *MET* mutation was identified in one patient. For the *BRAF* gene, two Val600Glu mutations were observed, and one patient presented with a *BRAF* amplification. An *ERBB2* exon 20 insertion was identified in one patient, and an *ERBB2* amplification was observed in six.

We compared the genomic alterations in our cohort with those reported in the Memorial Sloan Kettering Cancer Center (MSKCC) cohort accessed via cBioPortal [16]. Considering only NSCLC tumour tissue samples sequenced using the MSK Integrated Mutation Profiling of Actionable Cancer Targets (MSK‐IMPACT), the MSKCC cohort included 753 lung adenocarcinoma cases, 38 lung squamous cell carcinoma cases, and 89 classified as other subtypes. Our cohort demonstrated a similar percentage of alterations compared to MSKCC in TP53 (69% vs. 56%), KRAS (33% vs. 27%), ERBB2 (10% vs. 7%), ALK (10% vs. 9%), and BRAF (4% vs. 5%). However, EGFR (19% vs. 34%) and MET (6% vs. 13%) alterations were less frequent.

Pathogenic or likely pathogenic variants were more prevalent in SMARCA4 (14% vs. 9%), CHEK2 (11% vs. < 1%), PIK3CA (10% vs. 6%), and TET2 (10% vs. 2%), compared with the MSKCC cohort. Among CHEK2 variants, nine patients (82%) carried the Ile157Thr mutation, likely a germline variant. Eight were sequenced with F1CDx and one with F1LCDx, but as non‐tumour samples were unavailable, germline status could not be confirmed. TET2 is a tumour suppressor gene linked to various cancers, including haematological malignancies and solid tumours [17]. Among ten patients with truncating TET2 mutations, two were sequenced with F1LCDx and the rest with F1CDx. None had clonal haematopoiesis or haematological malignancies, suggesting these mutations were associated with the current lung cancer. Our cohort also had more RET (9% vs. 4%) and ROS1 (8% vs. 5%) alterations, although this was attributable to a higher proportion of variants of unknown significance.

The median tumour mutational burden (TMB) for the cohort was 5 mutations per megabase (mut/Mb), with a median of 7 mut/Mb (range 0–33) in F1CDx. The mean bTMB for the three F1LCDx assays was 1.33 mut/Mb (range 0–4). In total, 30 patients had a TMB of at least 10 mut/Mb (TMB‐H). Microsatellite instability was not detected in any of the F1CDx or F1LCDx assays.

**FIGURE 2:**
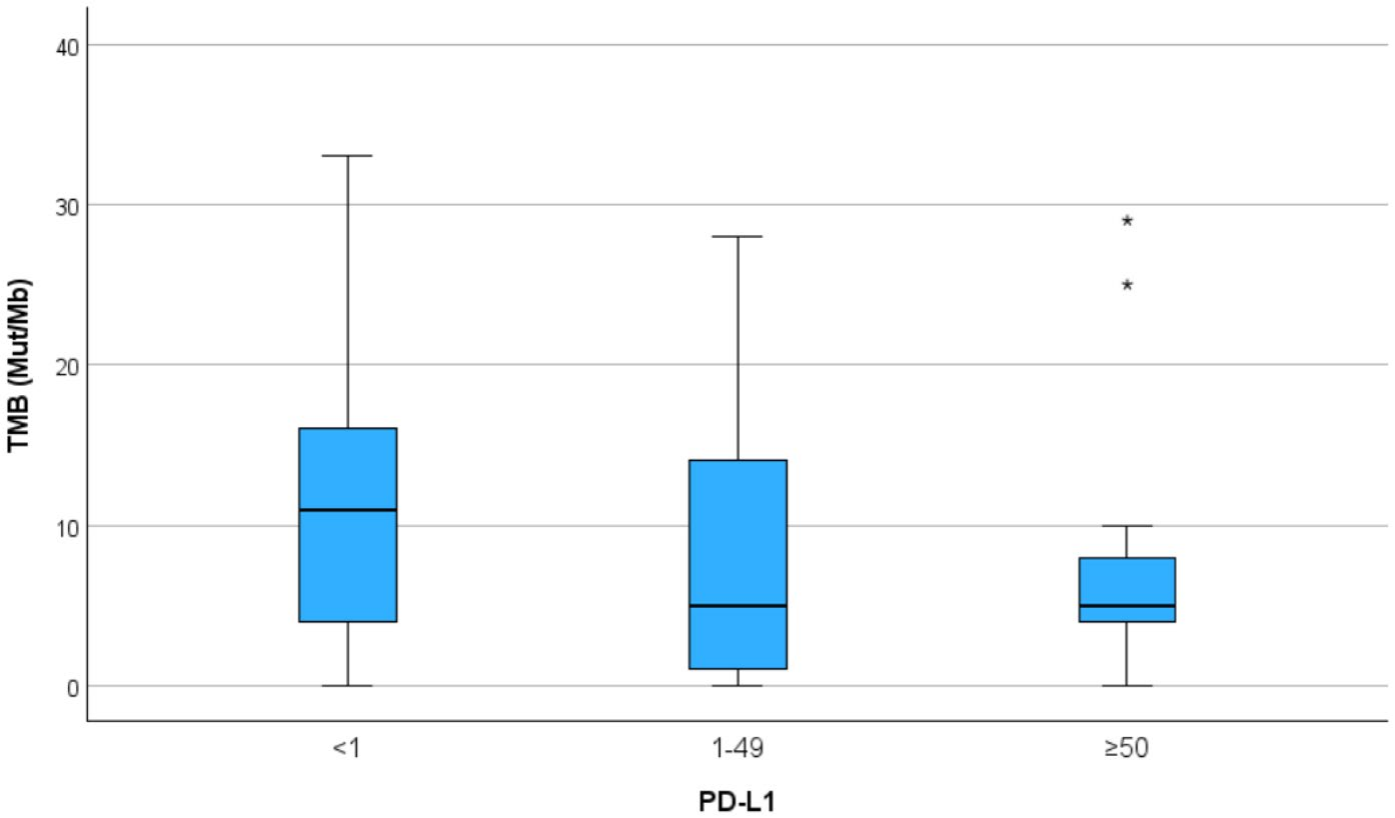
TMB across PD‐L1 expression subgroups.

PD‐L1 status was evaluated in 96% of patients. Patients were categorised into three clinically relevant PD‐L1 expression subgroups: less than 1%, 1%–49%, and 50% or higher, representing 32%, 34%, and 29% of patients, respectively. No correlation was observed between PD‐L1 expression and tumour mutational burden. A higher proportion of TMB‐H patients was found in the PD‐L1‐negative group (< 1%) compared with the PD‐L1 50% or higher group (42% vs. 11%, *p* = 0.001). TMB across PD‐L1 subgroups is displayed in Figure 2.

### 3.3 Co‐Occurring Mutations

*TP53* was the most prevalent co‐alteration occurring in tumours with *CDKN2A/B* alterations in 72% of patients (26/36), *KRAS* (72%, 23/32), *EGFR* (83%, 15/18), *FLT1* (75%, 12/16), *KEAP1* (65%, 11/17), *KMT2D* (70%, 14/20), *SMARCA4* (71%, 10/14), *STK11* (56%, 9/16), and in all tumours with *AKT3* alterations (100%, 8/8). Three patients with TKI‐sensitising *EGFR* alterations also had concurrent *TP53* mutations alongside alterations in tumour suppressor genes *ARID1A*, *NF1*, or *RB1*, which are associated with poorer outcomes [18]. *CDKN2A/B* co‐mutations were significantly represented in tumours with *STK11* (63%, 10/16, *p* = 0.024), *KEAP1* (59%, 10/17, *p* = 0.045), and *SPEN* (90%, 9/10, *p* < 0.001) alterations, and in all tumours with *MTAP* alterations (100%, 16/16, *p* < 0.001). Co‐occurring alterations with *KRAS* included *FLT1* (28%, 9/32, *p* = 0.033), *APC* (16%, 5/32, *p* = 0.015), and *KEAP1* (16%, 5/32), with four of these tumours also harbouring *STK11* alterations, forming a *KRAS‐KEAP1‐STK11* mutational profile (13%, 4/32). The *KRAS‐STK11‐KEAP1* combination is associated with worse overall survival [19].

### 3.4 ESCAT Actionability and Targeted Therapy

**FIGURE 3:**
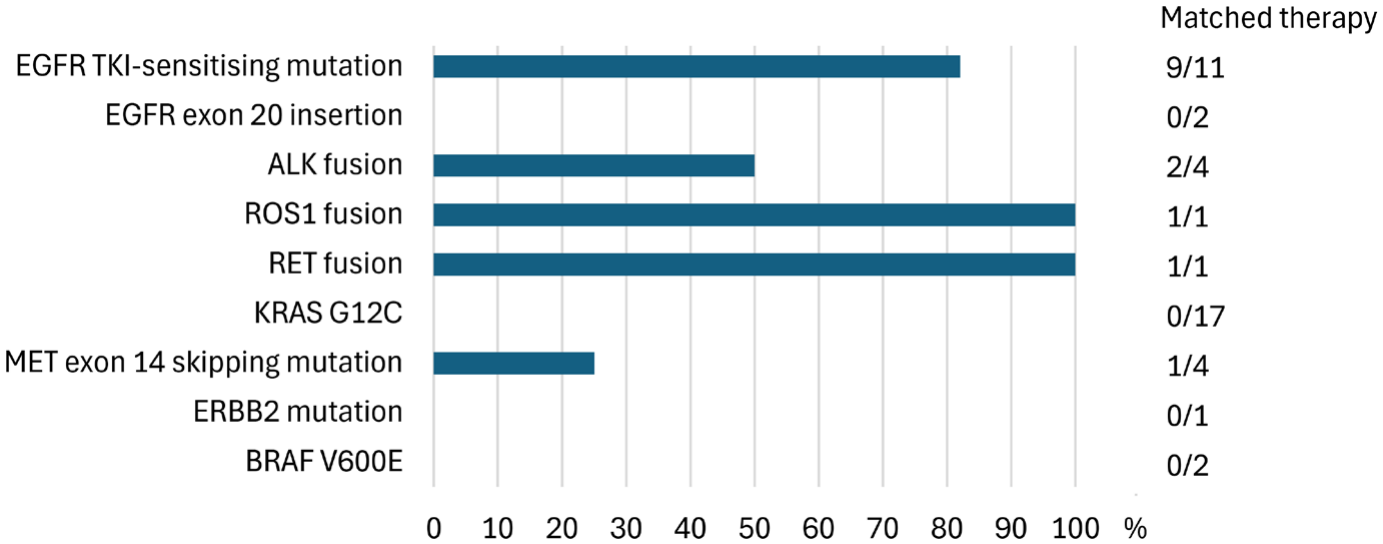
Genetic mutations identified throughout the cohort linked with matched therapies.

We classified the clinically actionable gene alterations using the ESMO Scale for Clinical Actionability of molecular Targets (ESCAT) [15]. A total of 43 patients (45%) harboured at least one actionable gene alteration with an EMA/FDA‐approved matching therapy: 42 patients had a level I alteration and one had a level II alteration. One third of these patients (33%) received the corresponding targeted therapy (Figure 3).

Patients with classical *EGFR* mutations were treated with osimertinib, while one with rare *EGFR* mutations received second‐generation *EGFR* inhibitors. Two patients with an *ALK* fusion were treated with alectinib, the patient with a *ROS1* fusion received crizotinib, and the patient with a *RET* fusion was treated with pralsetinib. One patient with a MET exon 14 skipping mutation received tepotinib following platinum‐based chemotherapy.

Two patients with *EGFR* TKI‐sensitising mutations and two with *ALK* fusions died before targeted therapy initiation. The primary barrier to receiving treatment was the lack of reimbursement by the Social Insurance Institution of Finland. Non‐reimbursed treatments included amivantamab (EGFR exon 20 insertions), sotorasib (KRAS Gly12Cys), trastuzumab deruxtecan (ERBB2 exon 20 insertions), and dabrafenib‐trametinib or encorafenib‐binimetinib (BRAF Val600Glu). Tepotinib was reimbursed for MET exon 14 skipping mutations post‐immunotherapy or chemotherapy, applicable to one patient in our cohort.

The FDA has approved the anti‐PD‐1 antibody pembrolizumab for metastatic solid tumours with a tumour mutation burden of ≥ 10 mut/Mb (TMB‐H), though EMA has not granted approval for pembrolizumab based on TMB‐H alone. TMB‐H is classified as an ESCAT level I alteration found in 30 patients (31%) within the cohort, ten of whom also had a level I gene alteration. In total, 63 patients (66%) had ESCAT level I or II genomic alterations.

### 3.5 Association of Clinical Characteristics on Genomic Alterations

Never‐smokers were more likely than ever‐smokers to harbour ESCAT level I EGFR alterations (37% vs. 8%, *p* = 0.004), while RET and ROS1 fusions were exclusive to never‐smokers. Excluding KRAS Gly12Cys, other ESCAT I/II gene alterations were more prevalent in never‐smokers than in ever‐smokers (58% vs. 18%, *p* < 0.001). Conversely, STK11 alterations were absent in never‐smokers (0% vs. 21%, *p* = 0.036). TMB correlated linearly with pack‐years of tobacco use (Spearman's coefficient 0.559, *p* < 0.001). Ever‐smokers had a median TMB of 8 mut/Mb compared with a median of 3 mut/Mb for never‐smokers. None of the never‐smokers exhibited a TMB of ≥ 10 mut/Mb (TMB‐H) (0% vs. 39%, *p* = 0.001). The comparison of actionable alterations between ever‐smokers and never‐smokers is displayed in Table 3.

**TABLE 3:** Actionable ESCAT level I/II alterations in ever‐smokers and never‐smokers.

Gene	Ever‐smokers*, *n* = 76 (79%)	Never‐smokers*, *n* = 19 (20%)
*ALK*	2 (3%)	1 (5%)
*ROS1*	0 (0%)	1 (5%)
*RET*	0 (0%)	1 (5%)
*EGFR*	6 (8%)	7 (37%) (*p* = 0.004)
*MET*	3 (4%)	1 (5%)
*BRAF*	2 (3%)	0 (0%)
*ERBB2*	1 (1%)	0 (0%)
*KRAS*	13 (17%)	1 (5%)
TMB‐H (≥ 10 mut/Mb)	30 (39%)	0 (0%) (*p* = 0.001)

* Smoking status was unavailable for one patient.

## 4 Discussion

We analysed comprehensive genomic profiling (GCP) results in Finnish NSCLC patients. CGP was intended for patients with advanced‐stage NSCLC, and the cohort included only four patients presenting with early‐stage disease. Consequently, the CGP findings predominantly reflect advanced‐stage NSCLC. Clinically actionable genetic alterations were identified in 45% of patients more frequently in never‐smokers (63% vs. 41%). However, only 33% of eligible patients received targeted therapy, largely due to lack of reimbursement. Significant inequities exist in access to cancer treatments across European countries [20]. While the time taken for cancer drugs to gain reimbursement approval following marketing authorisation is faster in Finland compared to the European average, it still takes nearly a year on average—351 days [21].

The most frequent actionable gene alteration in our cohort was KRAS Gly12Cys (17 patients), but none received targeted therapy due to non‐reimbursement. This alteration is more common in smokers [22] and was found in only one never‐smoker in our cohort. Other ESCAT I/II alterations were more prevalent in never‐smokers (*p* < 0.001). EGFR alterations, the second most frequent actionable change (13 patients), were significantly more common in never‐smokers (*p* = 0.004). Among patients with TKI‐sensitising EGFR alterations, 82% received targeted therapy. Two patients (18%) died before the treatment could be initiated.

EML4‐ALK fusions were detected in four patients, two of whom died before receiving the F1CDx results, illustrating the aggressive nature of the disease and emphasising the importance of timely detection, given the availability of effective therapies. Other actionable fusions included SDC4‐ROS1 and KIF5B‐RET, each identified in one patient, both of whom were never‐smokers. These patients received targeted therapy for their alteration.

The Finnish population exhibits genetic differences compared to other European populations, and there is limited data on the mutational landscape of Finnish NSCLC patients [9, 23]. This study is the first to describe the comprehensive genomic profiling of advanced‐stage NSCLC in this population. We identified nine patients (9.4%) with a CHEK2 Ile157Thr mutation. CHEK2 is a cell cycle control gene encoding a pluripotent kinase that can cause arrest or apoptosis in response to DNA damage [24]. Data from the Genome Aggregation Databases (gnomAD) v4.1.0 show a global prevalence of the CHEK2 Ile157Thr mutation of 4458/1614104 (0.28%) and 2614/1180010 (0.22%) in European non‐Finnish populations. The highest prevalence of the CHEK2 Ile157Thr mutation is indicated for the Finnish population at 1649/64022 (2.58%) [25]. Our results indicate a significantly higher prevalence of the CHEK2 Ile157Thr mutation among Finnish patients with advanced NSCLC compared to global, European non‐Finnish, and Finnish populations. The prevalence in our cohort also exceeds that of Finnish patients in the early‐stage lung adenocarcinoma cohort, where all CHEK2 alterations had a prevalence of 3.7% [9], and that of the MSKCC cohort of advanced NSCLC (< 1%) [16].

The CHEK2 Ile157Thr germline mutation has been linked to an increased risk of breast, colon, kidney, prostate, and thyroid cancers [26], but its association with lung cancer is less pronounced [24]. Some studies suggest it may confer protection against lung cancer [27], particularly in smokers and squamous cell carcinoma cases [28]. However, our findings contradict this, showing a significantly higher prevalence in advanced NSCLC, with no correlation to smoking or age.

Additional genomic differences were observed when comparing our cohort with the MSKCC advanced NSCLC cohort [16]. PIK3CA alterations were more prevalent in our cohort (10% vs. 6%). In NSCLC, *PIK3CA* alterations often co‐occur with other genomic changes, some of which are well‐established actionable genomic alterations, while *PIK3CA* itself is considered a potential passenger mutation [29]. In our cohort, PIK3CA was found alongside KRAS Gly13Asp, MET exon 14 skipping, and KIF5B‐RET fusion in three cases, but was present independently in most cases, suggesting a possible driver role [28].

Our cohort exhibited lower frequencies of EGFR (19% vs. 34%) and MET (6% vs. 13%) alterations compared to the MSKCC cohort [15], potentially due to differences in demographics. The MSKCC cohort consisted of 73% White, 17% Asian, and 10% other ethnic backgrounds, whereas our cohort included only one patient with an Asian immigrant background. Additionally, our cohort had a lower proportion of never‐smokers (19.8% vs. 45.6%) and female patients (35.4% vs. 59.4%) compared to the MSKCC cohort.

The most common co‐occurring alteration was TP53, observed in 83% of EGFR‐mutated tumours. TP53 mutations have been recognised as a negative predictive marker in patients receiving TKI treatment [30]. In our cohort, TP53 alterations co‐occurred with 73% of TKI‐sensitising EGFR mutations. Three of these cases also exhibited concurrent alterations in the tumour suppressor genes ARID1A, NF1, or RB1, suggesting even poorer prognosis and a need for more intensive follow‐up or more aggressive interventions beyond EGFR TKI monotherapy [18].

Tumour mutational burden (TMB) serves as a predictive biomarker for immune checkpoint inhibitor (ICI) therapy, as a high TMB increases the likelihood of a greater number of immunogenic neoantigens [31]. The FDA has approved pembrolizumab for metastatic solid tumours with TMB ≥ 10 mut/Mb, but large trials in NSCLC have not consistently demonstrated a survival benefit [32, 33]. Variability in sequencing panels and TMB thresholds limits its clinical utility [30]. A higher TMB threshold, closer to the 90th percentile, may be more effective in identifying patients benefiting from immunotherapy [33]. Ricciuti et al. also showed that increased TMB is associated with a clinical benefit from ICI therapy independent of PD‐L1 expression. In our cohort, the 90th percentile TMB was 22.5 mut/Mb, encompassing eight patients, four of whom exhibited negative PD‐L1 expression.

Lung cancers across all PD‐L1 expression levels may respond to ICIs, highlighting the need for additional biomarkers to predict immunotherapy efficacy [34]. A weak correlation between TMB and PD‐L1 expression has been reported in NSCLC [35], though it was not observed in our cohort. A higher proportion of TMB‐H patients was identified in the PD‐L1‐negative group (< 1%) compared to the PD‐L1 50% or higher group (42% vs. 11%, *p* = 0.001). Among various predictive biomarkers for immunotherapy, the combination of TMB and PD‐L1 IHC demonstrated the best balance between sensitivity and specificity. Integrating TMB with PD‐L1 IHC markedly improved sensitivity without compromising specificity [36].

Samples for NGS analysis were obtained from the most accessible site, with 54 (56%) taken from the primary tumour and 39 (41%) from metastatic sites. In addition, three liquid biopsy samples were collected from peripheral blood when tissue samples were inadequate or not available. Primary tumour samples were more often histological (69%), whereas samples from metastatic sites were more frequently cytological (68%), most commonly EBUS‐TBNA from metastatic lymph nodes. This reflects real‐world practice and the challenges of obtaining representative samples in NSCLC. No significant differences in tumour cell fraction were observed between biopsies, cytology specimens, or resection samples. A lower tumour cell fraction mainly reduces the sensitivity for detecting copy number alterations. No significant differences in genomic alterations were identified with respect to sample type or site of sampling.

This study has certain limitations, primarily its retrospective design and the relatively small patient cohort. In a real‐world setting, the samples obtained for NGS analysis were small in the majority of patients. Samples were taken from the primary tumour, a metastatic site, or a liquid biopsy from peripheral blood. While this could potentially influence the genomic findings, no such effect was observed in our study. Nevertheless, this study highlights the substantial practical relevance and real‐world impact of CGP on patient treatment. The cohort encompasses all patients from a single centre with advanced NSCLC, excluding those with squamous cell lung cancer and a substantial smoking history, who were considered eligible for cancer therapy over a period of more than one year. To our knowledge, this is the first study to report comprehensive genomic profiling in advanced‐stage NSCLC, providing valuable insights that significantly expand the limited data available on the Finnish population.

## 5 Conclusions

Comprehensive genomic profiling offers extensive information that can guide treatment decisions in NSCLC. In our cohort, 63% of never‐smokers and 41% of ever‐smokers were found to harbour ESCAT level I/II gene alterations eligible for EMA/FDA‐approved targeted therapies. CGP also provides insights into co‐occurring alterations that may influence treatment outcomes. Additionally, the tumour mutational burden (TMB) derived from CGP panels, alongside certain genomic profiles combined with PD‐L1 immunohistochemistry (IHC), can better predict the efficacy of immunotherapy, potentially helping to avoid ineffective treatments associated with adverse outcomes. Therefore, CGP has a significant impact on treatment strategies for the majority of NSCLC patients. However, in real‐world clinical practice, there are limitations to the practical application of this information, as optimal treatments may not always be accessible. A significant prevalence of the CHEK2 Ile157Thr mutation in Finnish NSCLC patients suggests a previously unreported association. Further studies are needed to validate these findings and assess their implications for cancer susceptibility assessment.

## Author Contributions

**Kirsi Hormalainen:** writing – original draft, writing – review and editing, conceptualisation, investigation. Kaisa Marttila: writing – original draft, investigation, visualisation. **Matti Nykter:** supervision. **Toomas Uibu:** writing – review and editing. **Jarkko Ahvonen:** writing – review and editing. **Vidal Fey:** visualisation. **Mauri Keinänen:** supervision. **Maarit Bärlund:** writing – review and editing, conceptualisation, supervision. **Arja Jukkola:** writing – review and editing, supervision.

## Conflicts of Interest

The authors declare no conflicts of interest.

## Data Availability

The data that support the findings of this study are available from the corresponding author upon reasonable request.
